# Seasonal Variation and the Effect of the Transition Period on Physical Fitness Parameters in Youth Female Soccer Players

**DOI:** 10.3390/sports12030084

**Published:** 2024-03-20

**Authors:** Koulla Parpa, Borko Katanic, Marcos Michaelides

**Affiliations:** 1 Faculty of Sports and Exercise Science, UCLan University of Cyprus, Avenue 12-14, Larnaca 7080, Cyprus; mmichaelides@uclan.ac.uk; 2 Montenegrin Sports Academy, 81000 Podgorica, Montenegro; borkokatanic@gmail.com

**Keywords:** sprint performance, jump, agility, maximal oxygen consumption

## Abstract

This study examined seasonal variation and the effect of the transition period on physical fitness parameters in female youth soccer players. Twenty-four players (13–17 years old) were assessed through field and laboratory testing. They completed laboratory testing three times: (1) at the beginning of the season (post-preseason), (2) at the end of the regular season (postseason), and (3) following the transition period (preseason). Field testing was conducted post-preseason and postseason. Results indicated that weight and body fat significantly increased from post-preseason to postseason and following the transition period. A paired samples T-test revealed that the players performed significantly better in the vertical jump and 30 m sprint test (all *p* < 0.01) postseason compared to post-preseason. Also, it was indicated that torque production for the right quadriceps, left quadriceps, and right hamstring (all *p* < 0.01) was significantly reduced after the transition period by 10%, 11.8%, and 10.5%, respectively. Cardiorespiratory measurements demonstrated that performance on an incremental cardiopulmonary treadmill test, maximal oxygen consumption, velocity at the first ventilatory threshold, velocity at the second ventilatory threshold, and velocity at maximal oxygen consumption (all *p* < 0.01) were significantly reduced postseason and following the transition period. Based on the results, coaches and trainers should primarily focus on maintaining the aerobic capacity of the players during the season, as it has been indicated to be reduced from the beginning to the end of the competitive period (VO2max reduced by 3.3%). In addition, they should focus on maintaining lower body strength and aerobic capacity during the transition period.

## 1. Introduction

Soccer is a high-intensity intermittent team sport that demands a variety of technical skills in addition to high levels of physical fitness [1]. It has been demonstrated that elite female soccer players cover a distance of 10.3 km during a competitive game, with high-intensity running accounting for 1.31 km [2]. In comparison, female youth players (15–17 years old) have been reported to cover 6.5 to 9.0 km during games, with positional differences comparable to elite women and high-intensity running ranging from 0.34 to 0.77 km [3]. In addition to high-intensity running, they perform a great number of accelerations, decelerations, and sprints [4]. Female players demonstrate high heart rates throughout a competitive game with periods of near maximum values [2]. Consequently, the ability to sustain high-intensity intermittent efforts requires aerobic fitness, anaerobic power, strength, speed, agility, and flexibility [5,6]. Research affirms that body fat percentage and high-speed knee flexor concentric strength are the most significant predictors of high-intensity and very-high-intensity running [7]. At the same time, variables associated with aerobic capacity are considered significant predictors of total distance and low-intensity running for female soccer players participating at a national level [7]. Moreover, high-intensity actions such as sprinting, changing of direction, and jump performance have been indicated as discriminatory variables in female soccer players of different levels [8,9]. Even though junior female players demonstrate lower levels of anaerobic power and aerobic capacity than senior players [9], it would appear as reasonable that the aforementioned fitness parameters are essential physical determinants during a soccer game and should be trained intensively regardless of age and competitive level [9,10,11]. 

The assessment of players’ physical fitness through field and laboratory testing during the season, as well as after the transition period, is of great importance not only for the identification of strengths and weaknesses of the soccer players but also for the evaluation of the training programs and the changes in fitness status across the various phases of the season [12,13]. The seasonal variation in physical fitness has been investigated extensively in male elite soccer players, semi-professional players, and youths [14,15,16,17]. While some authors indicated that in elite male soccer players, aerobic performance parameters remain relatively constant throughout the competitive period [14], others suggested significant decreases in aerobic fitness from midseason to the end of the season [16]. A considerable improvement was indicated in the vertical jump, sprint, agility, flexibility, and aerobic capacity from preseason to midseason, with no further changes from midseason onward [15,16]. In a different study, sprint ability continued to improve until the end of the competitive season [18].

Regarding the transition period, it has been indicated to negatively impact body composition, muscle power, sprinting performance, maximal oxygen consumption, time to exhaustion, and intermittent running performance in male soccer players [19]. Also, a transition period of six weeks resulted in significant adverse changes in the sprint performance, countermovement jump, squat jump, and body composition in Greek professional soccer players [20]. Similarly, a study on elite female soccer players indicated reductions in VO2max, running time on the treadmill, and quadriceps torque following a 4-week transition period with concurrent increases in body weight and body fat percentage [21]. On the contrary, a recent study indicated that highly trained soccer players were not only able to maintain satisfactory levels of performance but also some strength and power parameters improved significantly after the transition period [22]. 

In addition to some inconsistencies evident in the male studies, only a limited number of studies were conducted on the seasonal variation in physical fitness in female soccer players. Research on Polish female soccer players demonstrated improvements in the countermovement jump and functional movement screening during the in-season period [23]. An additional study on female youth players indicated that acceleration, sprint performance, and change of direction decreased throughout the season, while repeated sprint ability improved in U13 players [24]. Lastly, research indicated that in female soccer players under 16 years of age, the relative strength, countermovement jump, 10 m sprint, 30 m sprint, and change of direction improved from preseason to postseason [25]. 

To the best of our knowledge, the limited studies on female youth soccer players examined only a few fitness components using field testing. Therefore, the assessment of seasonal variation in fitness parameters of female youth soccer players using laboratory and field testing has yet to be investigated. At the same time, none of the studies on female youth players evaluated the effect of the transition period on physical fitness parameters. This study aimed to examine (1) body composition and physical fitness parameters assessed through laboratory and field assessments over the course of an entire soccer season (post-preseason and competition period) and (2) evaluate the effect of the transition period on body composition, cardiovascular fitness, and lower body strength (assessed through isokinetic testing). We hypothesize that there would be no significant changes in physical fitness parameters and body composition from the beginning of the season (post-preseason) to the end of the regular season (postseason). Also, it is hypothesized that there would be no significant changes in body composition and physical fitness parameters assessed through laboratory testing following the transition period. 

## 2. Materials and Methods

### 2.1. Participants 

A total of 24 female youth (13–17 years old) soccer players from the same team participated in this study. Their physical fitness was assessed through field and laboratory testing. All the participants had prior experience with the testing procedures, and participation in the study was voluntary. Legal guardians and players were informed of the risks and processes, and parents/legal guardians signed an informed consent form before any testing was carried out. Ethical guidelines were followed according to the Helsinki Declaration, and the study was approved by the National Committee of Bioethics. Female players who reported musculoskeletal injuries in the past six months or throughout the season were excluded from the study. In addition to the musculoskeletal injuries, the study did not include players who did not participate in the regular training sessions. All players participated in the same in-season training program, which was recorded and supervised and included four weekly training sessions and one official game. A sample of typical in-season training is provided in Table 1. Their physical activities during the transition period were not recorded or supervised, but they were instructed to follow a training regimen that their fitness coach provided in order to maintain their physical fitness and muscular strength. During the first week of the transition period, the players abstained from any physical activities, while during the following four weeks, they had to complete two strength/power training sessions per week and two cardiovascular sessions on alternate days. Specifically, they were instructed to complete a strength/power training session on Monday and Wednesday, while cardiovascular training was recommended on Tuesday (continued running) and Friday (speed intervals). None of the players reported any injuries during the season. The team finished second in the McDonald’s Championship-Amazons and received first place in the 1st Girls’ National Soccer Tournament in 2022. 

### 2.2. Experimental Design

**Figure 1:**
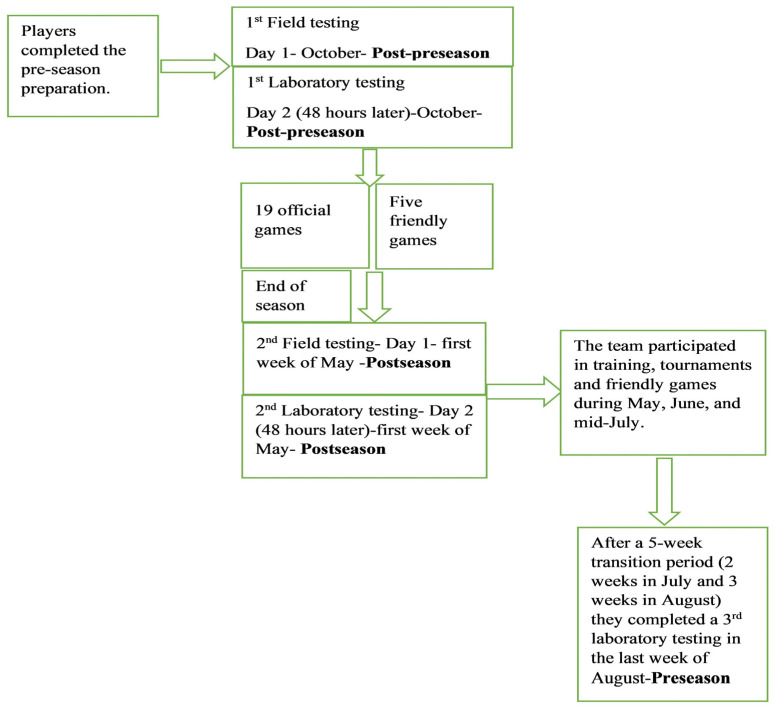
Experimental design.

All participants completed laboratory testing at three time points: (1) the beginning of the season (post-preseason), (2) the end of the regular season (postseason), and (3) following the transition period (preseason). In addition, field testing was conducted twice (post-preseason and postseason). The experimental protocol is presented in Figure 1. Laboratory and field testing were separated by at least 48 h. A standard field warm-up, which included jogging and dynamic stretching, was completed before each field testing, while a 5 min warm-up on the bike was completed before laboratory testing. The players were instructed to abstain from heavy physical activities the day before the testing, and the measurements were obtained from 15:00 to 19:00 during their regular training hours. Before each test, the same investigator demonstrated the correct test technique, and the same two investigators carried out all the testing. The order of the testing was the same at all testing times. On the first day, the players completed the field testing, which included the vertical jump test, the 30 m sprint, and agility T-testing. On the second day of the testing (48 h later), the players underwent an anthropometric evaluation (weight, height, and body fat), which was followed by laboratory testing (flexibility, isokinetic assessment, and cardiopulmonary testing on the treadmill). 

### 2.3. Field Tests

Before the field testing, the players were instructed to follow a typical 15 min field warm-up, which included jogging (submaximal running) and dynamic stretching. The warm-up also included sidestepping, knee lifting, heel flicks, and lunge strides. 

### 2.4. Vertical Jump

Jump height was measured using a Vertec (JUMPUSA.com, Sunnyvale, CA, USA). Following verbal explanation and demonstration, the players’ standing reach height was measured, and the Vertec device was adjusted accordingly. The Vertec device is comprised of plastic vanes arranged in 1.25 cm increments and attached to a metal pole. The players had to use their dominant hand to displace the highest possible vein with an overheard arm swing. They performed three vertical jumps, and the number of displaced Vertec vanes was counted to calculate the vertical jump. The rest time between the jumps was 30 s. Forward, backward, and sideways jumping were not allowed. Thus, the players were instructed to jump vertically, from a standing position (free arms), as high as possible and land on the same spot. The best of the three jumps was used for a statistical analysis. Intra-class correlation coefficients were high between the three trials (r = 0.99, *p* < 0.001). 

### 2.5. 30-m Sprint Test

Sprint performance was assessed using a maximal 30 m sprint according to the methods described by previous investigators [26]. Sprint times were recorded at 10, 20, and 30 m, but only the 30 m sprinting time was used for the analysis. All players began in a standing position, with the front foot positioned 0.5 m from the first timing gate. The time at 10, 20, and 30 m was automatically recorded using photocell gates (accuracy of 0.01 s; Brower Timing Systems, Salt Lake City, UT, USA) placed 0.4 m above the ground. The players performed two trials with a 2 min rest between the trials, and the best 30 m sprinting time was recorded for the statistical analysis. The intra-class correlation coefficient was high between the two trials (r = 0.98, *p* < 0.001). 

### 2.6. Agility T-Test

The players’ agility was evaluated with the T-test, which involves forward, backward, and lateral running. The test required four cones arranged into a T-shape according to the methods described by previous investigators [26]. The first and second cones were placed 9.14 m apart, while cones three and four were placed 4.57 m from either side of the second cone. The first cone (starting cone) was placed between two photocell gates (2 m apart). The players were instructed to sprint forward from the first to the second cone (for 9.14 m), then right shuffle to the third cone (for 4.57 m), then left shuffle to the fourth cone (for 9.14 m), then go back to the middle cone before performing backward running to the start line (cone 1). If the players failed to touch the designated cone or failed to face forward at all times, the test was considered invalid. The timing gate at the start–finish line recorded the total time needed to complete the test. The players performed the test twice, and the best time was recorded for a statistical analysis. The intra-class correlation coefficient was high between the two trials (r = 0.97, *p* < 0.001). 

### 2.7. Laboratory Testing 

Before the tests, players were instructed to have a 5-min warm-up on a mechanically braked cycle ergometer. The tests were conducted in the order presented below, with at least a three-minute rest period between each test. 

### 2.8. Anthropometric Measurements

Stature was recorded (to the nearest 0.1 cm) using a wall stadiometer (The Leicester Height Measure, Tanita, Tokyo, Japan). A leg-to-leg bioelectrical impedance analyzer system (BC 418 MA, Tanita, Tokyo, Japan) was used to measure body composition using the athlete mode and the input variables of body height, gender, and age. The players were instructed to follow the standard guidelines before the bioelectrical impedance testing, which included fasting for at least four hours, abstaining from intense physical activity the day before the testing, abstaining from drinks with high caffeine content in the previous twelve hours, and emptying their bladder before the test. The same experienced investigator performed the same procedure throughout the three time points (post-preseason, postseason, and preseason).

### 2.9. Sit and Reach Test

A custom box (32.4 cm high and 53.3 cm long) with a 26 cm heel line mark was used to assess the flexibility of the hamstrings and lower back muscles. The players placed the soles of their feet (barefoot) against the box with their knees fully extended. They had to lean forward with palms facing downward and one hand on top of the other. They were instructed to learn forward as far as possible in a slow-paced and well-controlled movement. They performed the test three times, and the best trial was recorded for the analysis. 

### 2.10. Isokinetic Strength Measurements

Isokinetic knee strength was computed utilizing a Humac Norm and Rehabilitation device (CSMI, Stoughton, MA, USA) according to the methodology described by previous investigators [21]. Once the players were appropriately positioned on the isokinetic device, they performed five sub-maximal concentric knee flexion and extension repetitions for familiarization purposes. The isokinetic testing included three maximal flexion and extension repetitions at an angle speed of 60°/s. The peak concentric torque (Nm) out of the three repetitions of the quadriceps and hamstring muscles was retained for an analysis. Hamstring-to-quadriceps ratios (H/Q) and interlimb deficits (Q-Q and H-H) were also recorded. 

### 2.11. Cardiopulmonary Exercise Testing

The players completed incremental maximal cardiopulmonary testing on a treadmill (h/p/Cosmos Quasar med, H-P-Cosmos Sports and Medical GmbH, Nussdorf-Traunstein, Germany) according to the methodology described by previous investigators [27]. During the test, the inclination was kept constant at 1% [28]. The players started the test at a speed of 6 km/h, which was increased every 3.15 min by 2 km/h. The test was terminated when the players reached volitional fatigue. A breath-by-breath analysis was performed on a Cosmed Quark CPET system (Rome, Italy) under constant laboratory conditions (temperature at 22 ± 1 °C; relative humidity at 50%). The data were filtered for an average of 10 s for the highest value of the VO2max to be determined. The first ventilatory threshold point (VT1) was determined through the V-slope method and verified at the nadir of the VE/V O_2_ curve, while the second ventilatory threshold (VT2) was detected at the nadir of the VE/V CO_2_ curve. VT1 and VT2 were detected by the software, but the points were verified manually by two experienced researchers. 

### 2.12. Statistical Analyses 

All statistical analyses were performed in IBM^®^ SPSS^®^ Statistics, version 28.0, for Windows (SPSS Inc., Chicago, IL, USA). The normality assumption was assessed with the Shapiro–Wilk test (*p* > 0.05). All parameters are presented as the mean and standard deviations, as the normality was confirmed. One-way repeated measures ANOVA was used to determine the differences in the dependent variables (flexibility, endurance, lower body strength) based on the three testing times. In cases where Mauchly’s test of sphericity was violated, the Greenhouse–Geisser values were used. One-way repeated measures ANOVA was followed by pairwise comparisons to identify the differences among all possible pairs. The Bonferroni post hoc method was used to perform pairwise comparisons between group means. The effect size was estimated with partial eta-squared (η^2^). For the field measurements that were obtained only at the beginning and the end of the regular season, a paired sample T-test was utilized. Cohen’s d was calculated to determine the effect size (ES) and present the magnitude of the reported effects. The level of significance was set at *p* < 0.05.

## 3. Results

The anthropometric characteristics, body composition, and flexibility of the female soccer players are presented in Table 2. Results indicated that weight [F (1.52, 34.94) = 114.36, *p* < 0.01, partial η^2^ = 0.83] and percent body fat [F (1.43, 32.88) = 102.55, *p* < 0.01, partial η^2^ = 0.82] were significantly different during the three testing points. The values of partial eta-squared indicated that 83% and 82% of the variance in weight and body fat, respectively, could be attributed to the different testing times. In addition, flexibility has been demonstrated to be significantly greater at the end of the regular season. In contrast, no significant differences in flexibility were evident between the beginning of the season and following the transition period.

The results of the vertical jump, 30 m sprint test, and agility T-test are presented in Table 3. Based on the paired samples T-test, the players performed significantly better in the vertical jump [t (23) = −3.12, d = 0.20, *p* < 0.01] and 30 m sprint test [t (23) = 9.46, d = 1.02, *p* < 0.01] at the end of the regular season compared to the beginning of the season. At the same time, no significant changes were observed in the T-test for agility. 

Based on the isokinetic measurements (Table 4), it was indicated that the torque production at 60°/sec for the right quadriceps [F (2, 38) = 22.14, *p* < 0.01, partial η^2^ = 0.54], left quadriceps [F (2, 38) = 14.02, *p* < 0.01, partial η^2^ = 0.43], and right hamstring [F (2, 38) = 11.96, *p* < 0.01, partial η^2^ = 0.39] was significantly different between the three testing times. The left hamstring indicated a borderline significant difference in torque production between the three testing times [F (2, 38) = 2.98, *p* = 0.06, partial η^2^ = 0.14]. No significant changes were observed between the three testing times for the hamstring-to-quadriceps ratio percentiles or hamstrings and quadriceps deficits. 

Based on the cardiorespiratory measurements (Table 5), it was indicated that running time on the treadmill [F (2, 32) = 35.37, *p* < 0.01, partial η^2^ = 0.69], VO2max [F (2, 32) = 19.70, *p* < 0.01, partial η^2^ = 0.55], the velocity at the first ventilatory threshold (LT) [F (2, 32) = 11.01, *p* < 0.01, partial η^2^ = 0.41], the velocity at the second ventilatory threshold [F (2, 32) = 9.82, *p* < 0.01, partial η^2^ = 0.38], and the velocity at VO2max [F (2, 32) = 28.51, *p* < 0.01, partial η^2^ = 0.64] were significantly different based on the three testing times.

## 4. Discussion

This study aimed to examine the seasonal variation and the effect of the transition period on physical fitness parameters in female youth soccer players. The main findings indicated that height, body weight, and body fat increased throughout the season and following the transition period. Field testing demonstrated improvements in the vertical jump and 30 m sprint time from post-preseason to postseason, while no changes were shown in the agility T-test. Laboratory testing revealed no changes in the quadriceps and hamstring strength from the beginning to the end of the season, while significant reductions in strength were evident after the transition period. Finally, the 5-week transition period resulted in significant adverse changes in run time on the treadmill, VO2max, and velocities at VT1 and VT2. Reductions in the aforementioned cardiorespiratory variables were also evident from post-preseason to postseason, in addition to a significant decrease in the velocity at VO2max. 

Insight into anthropometric characteristics reveals that the average values of height and body weight of our players at the initial measurement approximately correspond to female youth soccer players from Spain [29] and England [24,25] in the given age group, while slightly lower values were indicated compared to female soccer players from Germany [30]. However, these height and weight values correspond to the review study by Malina, Martinho, Valente-dos-Santos, Coelho-e-Silva, and Kozieł (2021) [31], which classified height and weight of female youth players by age based on 35 studies. However, when analyzing the percentage of body fat of our players, it is noticeable that there are significantly higher average values (21.39–24.05%) compared to female soccer players of the same age, whose values ranged from 14.7 to 18.5% at most [29,30]. Nonetheless, it should be emphasized that our anthropometric values do not deviate much from the reference values of senior professional female soccer players, ranging from 1.61 to 1.70 cm for height, 56 to 65 kg for weight, and 14.6 to 20.1% for %BF [32]. 

Regarding the body composition changes, our study indicated an increase in height, body fat, and body weight from the beginning to the end of the season, with the greatest increase occurring after the transition period (Table 2). These results are consistent with other studies [25,29,30], where linear growth in height and weight was also recorded among female youth soccer players during the season. It is known that physical growth in young girls can occur until the age of 17 [33], until estrogen levels rise and epiphyseal fusion begins, ultimately leading to the cessation of height growth [34]. This is precisely the reason for the physical growth observed in our players at this age. As for the percentage of body fat, similar to our study, Spanish players also showed a significant increase by the end of the season [29], while in the study by Lesinsky et al. (2017) [30], the percentage of body fat in German female youth soccer players varied throughout the season, with authors attributing it to different training goals during specific periods of the season. In the absence of studies examining seasonal changes in body fat among female youth soccer players, we can attempt to compare these findings with those of older girls. Our results are in agreement with a study that examined the body composition of National Collegiate Athletic Association Division 1 female soccer players through the competitive seasons, which indicated an increase in body fat at the end of the season compared to in-season [35]. However, these results are difficult to compare with those of elite female players as biological factors and female hormones at the initiation of puberty may change the body’s proportion of lean, fat, and skeletal mass [36]. Despite the changes in female hormones, the increase in body fat was more evident following the transition period, which agrees with previously published studies on female soccer players [21]. While detraining is typically observed in adult soccer players, it is more complex in younger players and reported changes in body composition could be multifactorial and likely attributed to training goals, competition, nutrition, growth, and hormonal changes. Nonetheless, attention should be paid to the body composition of female youth soccer players, as it is a significant factor strongly linked to soccer performance [3], particularly focusing on subcutaneous fat, which acts as unnecessary weight during locomotion and makes a difference between elite and lower-ranked players [37]. This is in line with findings by Ostojić [18], indicating that a reduction in body fat percentage during the season improves sprinting performance in professional soccer players, emphasizing why this parameter is of particular importance.

Flexibility has been demonstrated to be significantly greater from post-preseason to postseason. In contrast, no significant differences in flexibility were evident between post-preseason and following the transition period (Table 2). These findings are in agreement with studies on male soccer players that indicate an improvement in flexibility during the in-season period, with the lowest flexibility in the preseason [15,16], but contrary to the study by Rup and Kupig (1995) [38] who found that hamstring flexibility decreases as the season progresses. This should be further investigated, especially since no study has examined changes in flexibility among female soccer players, both younger and senior players.

Field testing (Table 3) revealed that the players performed significantly better in the vertical jump (40.14 ± 6.46 cm versus 41.43 ± 6.32 cm) and 30 m sprint test (5.25 ± 0.28 s versus 4.97 ± 0.27 s) at the end of the regular season compared to the beginning of the season. Sprint times (30 m) of our players at the end of the season were comparable to those presented by other investigators in a study on English peers (4.91–5.04) [25], as well as on Danish top league players (5.06 ± 0.06 s) [39]. Improvements in the vertical jump and sprint times from preseason to midseason were indicated in studies on male soccer players [15,16], with some researchers suggesting improvements in sprint ability until the end of the competitive period [18]. Similar improvements in jump performance during the in-season period were shown in women soccer players [23] and youth players [25]. However, it should be noted that contrary to these results, some studies reported no change in speed and agility [30], and some studies even indicated a decrease in sprint performances throughout the season [24], which could be explained by the cumulative effect of fatigue during the season due to a large number of games, as well as a shift in training focus to specific tactical tasks. However, this cannot be applied to our players, as they are youth players whose goal is still player development rather than competition, indicating that our players improved their performance during the training and competitive period. On the other hand, the weak results in the initial measurement after the transition period clearly reflect insufficient training during that period, leading to a decline in performance. Numerous studies have indicated the adverse effects of detraining or insufficient training on body composition and performance in soccer players [20,40], where even six weeks of insufficient training lead to a significant increase in body weight and %BF, with a decrease in performance in soccer players [20].

When evaluating peak concentric torque of the quadriceps and hamstring muscles, this study indicated that the torque production did not change from the beginning of the season (post-preseason) to the postseason period (Table 4), suggesting that the players managed to maintain lower body strength during the in-season period. On the contrary, the peak torque of the quadriceps and hamstring muscle group was significantly reduced following the 5-week transition period (Table 4). No studies were identified that examined the peak isokinetic torque of female youth soccer players during the season, whilst our results agree with a study on female soccer players, which indicated reductions in peak isokinetic torque of the quadriceps muscles after a 4-week transition period [21]. A similar study [25] investigating maximum lower limb force on a force platform found that the highest peak force (PF) of female youth soccer players from England during the season was significantly higher compared to preseason, which aligns with our results. It is well known that lower limb strength is crucial for success in soccer, as players need to generate high forces for kicking, tackling, turning, and jumping [9]. Therefore, players during the transition period need to engage in specific physical activities to avoid the detraining consequences that affect muscle strength reduction and other performances [19].

When it comes to cardiorespiratory parameters, it should be noted that VO2max is considered the best indicator of cardiorespiratory system functionality [41] and correlates with the total distance covered by soccer players during a game [42], as well as with high-intensity running in soccer [2,43]. In our study, it was observed that the average VO2max values of our players were slightly higher than the results of Spanish female soccer players of the same age group [29]. Based on the comprehensive study by Datson et al. (2014) [32], who documented VO2max values ranging from 49 to 57 mL/kg*min for senior female soccer players based on 15 recent studies, it can be noted that the lower range of these values would correspond to our parameters, considering that we are dealing with youth female soccer players. Our study revealed noteworthy decrements in VO2max, treadmill running time, and velocities at VT1, VT2, and VO2max from post-preseason to postseason (Table 5) with a 3.3% reduction in VO2max, 9.6% reduction in the running time on the treadmill, 9.9% reduction in the velocity at VT1, 8.2% reduction in the velocity at VT2, and 12.5% reduction in the velocity at VO2max. Additionally, these parameters were further reduced following the transition period with an additional decrease of 1.8% in VO2max and an 8.5% reduction in the running time on the treadmill. Furthermore, the velocities at VT1 and VT2 were reduced by 4.1% and 3.3%, respectively, while the velocity at VO2max remained unchanged from postseason to preseason (following the transition period). These results do not align with a study on Spanish peers [29], where there was no significant difference in VO2max between different periods in the season, nor with senior players who performed the YoYo aerobic test [44]. These conflicting results may indicate the implementation of a specific conditioning program for these players outside of the season. However, similar to our findings, research affirms a negative impact of the transition period on maximal oxygen consumption, time to exhaustion, and running time on the treadmill on both female [21] and male soccer players [19,45], along with lower VO2 at the anaerobic threshold [46]. Some research on soccer players suggests that after just eight weeks of inadequate training, players’ VO2max is significantly reduced [16]. Therefore, it is crucial for players to maintain their aerobic systems as they play a vital role in players’ performance [47]. It has also been found that players with a more efficient aerobic system can maintain a higher intensity during soccer games [48] and recover faster between high-intensity sprints [49]. 

The reductions in aerobic capacity from post-preseason to postseason could be attributed to the accumulative effect of fatigue across the season or the change in the training load or focus. It is well known that players focus primarily on physical performance during the preseason preparation [13], while during the season, the focus may shift to technical and tactical training. Considering that this study examined the players after the preseason preparation, comparing the preseason preparation training to the typical in-season training program is impossible. Thus, whether the decrease in aerobic performance was due to the accumulative effect of fatigue or a change in training focus/load cannot be confirmed, which is considered a study limitation. 

Generally, the transitional period had a negative impact on the performance of female soccer players. This negative influence relates to body composition, muscle strength, and the cardiorespiratory system, which is consistent with studies on male soccer players [19]. In contrast, numerous studies have shown that players who followed a training plan during the transitional period were able to maintain their performance levels [50,51]. In this regard, these results highlight the need to propose an individualized off-season supervised training plan, which would help young players maintain their strength and aerobic capacity, enabling them to enter the preparatory period in a better physical condition. 

### Limitations and Future Suggestions

One of the main limitations of this research is the lack of an aged-matched control group, which could have helped verify some improvements (sprint and jump performance) that might have been due to normal growth or maturation. An additional limitation is the lack of testing during the midseason, which might have helped identify when aerobic capacity was reduced during the season. Also, the fact that the players’ physical activity during the transition period was not recorded or supervised is considered a limitation as it may explain the changes in physical fitness and body composition. Lastly, the study did not control for hormonal fluctuations or the nutritional intake of the players. Therefore, suggestions for future research include incorporating a control group; covering more time points for testing (beginning of preparation, end of preparation, midseason, end of season); forming a subsample according to playing position in the team; creating a comprehensive seasonal training protocol for youth female soccer players. 

## 5. Conclusions

Despite the mentioned limitations, this study contributes significantly to understanding seasonal changes in physical parameters among female youth soccer players. It is one of the few studies that have examined seasonal changes in physical fitness parameters among female youth soccer players, encompassing a significant number of parameters, including anthropometry, sprint and jump performance, agility, flexibility, lower extremity muscle strength, and cardiorespiratory function. The study yielded several significant findings: (i) height, body weight, and body fat of the players increased throughout the entire season and after the transitional period; (ii) improvements in the vertical jump and 30 m sprint time from post-preseason to postseason; (iii) significant reductions in maximal quadriceps and hamstring force (torque) after the transition period; (iv) significant negative changes in treadmill running time, VO2max, and velocities at VT1, VT2, and VO2max from post-preseason to postseason and following the transition period.

Our results may aid coaches and fitness professionals in creating and implementing an organized training regimen for female youth soccer players across the season and during the transition period. This approach aims to maintain body composition, lower body muscular strength, and aerobic capacity [20].

## Figures and Tables

**Table 1:** Typical in-season weekly training.

Monday	Tuesday	Wednesday	Thursday	Friday	Saturday	Sunday
Main areas: recovery/high-intensity transition (~45 min to 1 h)	Main areas: transition to attack (~90 min)	Main areas: transition to attack (~85–90 min)	Day off	Main areas: crossing and finishing (~60 min)	Game day	Day off
Warm-up: 20 min of passing drills with a partner at low intensity	Warm-up: 10 min	Warm-up: 20 min		Warm-up: 15 min		
Rondo: 6 min (2 teams)	Fitness and strength training: 25 min	Speed, agility, and shooting drills: 10 min		Rondo: 8 min (2 teams)		
Phase of the game: 15 min with some defensive drills (11 versus 11) at low intensity	Defense drills: 15 min of tackling, marking, and positioning	Phase of the game: 20 min (11 versus 11)		Small-sided game: 12 min, three teams (4 versus 4 with 2 goalkeepers)		
Four sprint drills	Phase of the game: 15 min of small-sided game. Regular game: 12 min	Regular game: 20 min in a large field		Crossing and finishing: 10 min		
Cool down: 15 min of stretching	Cool down: 15 min of stretching	Cool down: 15 min of stretching		Cool down: 15 min of stretching		

**Table 2:** Anthropometric characteristics, body composition, and flexibility of the players at the beginning of the season (post-preseason), end of the regular season (postseason), and following the transition period (preseason) (Mean ± SD).

Parameter	Post-Preseason (1)	Postseason (2)	Preseason (3)	Repeated Measures ANOVA		
	(n = 24)	(n = 24)	(n = 24)		Mean Difference	Significance
Age (years)	14.6 ± 0.9	15.5 ± 0.9	15.6 ± 0.9	1–2	−0.92 *	<0.01
				2–3	−0.08	0.06
				1–3	−1.00 *	<0.01
Weight (kg)	52.16 ± 5.18	53.63 ± 5.44	55.18 ± 5.59	1–2	−1.47 *	<0.01
				2–3	−1.55 *	<0.01
				1–3	−3.02 *	<0.01
Height (cm)	160.1 ± 5.1	160.9 ± 5.0	161.4 ± 5.2	1–2	−0.12 *	<0.01
				2–3	−0.46 *	<0.01
				1–3	−1.27 *	<0.01
Body Fat %	21.39 ± 3.59	22.48 ± 3.92	24.05 ± 4.42	1–2	−1.09 *	<0.01
				2–3	−1.56 *	<0.01
				1–3	−2.65 *	<0.01
Sit and Reach (cm)	34.37 ± 8.33	35.29 ± 8.30	34.17 ± 8.53	1–2	−0.92 *	<0.01
				2–3	1.13 *	<0.01
				1–3	−0.21	0.10

Note: * *p* < 0.05.

**Table 3:** Vertical jump, 30 m sprint test, and agility T-test recorded at the beginning of the season (post-preseason) and the end of the regular season (postseason) (Mean ± SD).

Parameter	Post-Preseason	Postseason	95% CI of the Difference		Cohen’s d
	(n = 24)	(n = 24)	Lower	Upper	
Vertical jump (cm)	40.14 ± 6.46	41.42 ± 6.39 *	−2.08	−0.48	0.20
30 m sprint time (s)	5.25 ± 0.28	4.97 ± 0.27 *	0.22	0.35	1.02
T-test (s)	11.95 ± 0.86	11.90 ± 0.73	−0.12	0.22	

Note: * *p* < 0.05.

**Table 4:** Isokinetic torque measurements (60°/sec) of the players at the beginning of the season (post-preseason), end of the regular season (postseason), and following the transition period (preseason) (Mean ± SD).

Parameter	Post-Preseason (1)	Postseason (2)	Preseason (3)	Repeated Measures ANOVA		
	(n = 20)	(n = 20)	(n = 20)		Mean Difference	Significance
RQ (Nm)	124.25 ± 15.84	124.65 ± 17.07	112.05 ± 16.71	1–2	−0.40	0.87
				2–3	12.60 *	<0.01
				1–3	12.20 *	<0.01
LQ (Nm)	119.60 ± 19.96	120.75 ± 19.31	106.45 ± 16.73	1–2	−1.15	0.74
				2–3	14.30 *	<0.01
				1–3	13.15 *	<0.01
RH (Nm)	78.15 ± 12.26	78.25 ± 11.25	70.05 ± 11.01	1–2	−0.10	0.96
				2–3	8.20 *	<0.01
				1–3	8.10 *	<0.01
LH (Nm)	81.45 ± 12.59	81.50 ± 12.45	76.20 ± 9.58	1–2	−0.05	0.98
				2–3	5.30 *	0.05
				1–3	5.25	0.06
Deficit Q (%)	6.75 ± 6.30	6.30 ± 6.11	7.20 ± 6.70	1–2	0.45	0.78
				2–3	−0.90	0.56
				1–3	−0.45	0.81
Deficit H (%)	7.95 ± 4.89	8.65 ± 7.34	10.30 ± 5.99	1–2	−0.70	0.62
				2–3	−1.65	0.35
				1–3	−2.35	0.07
HQR % Right Side	63.25 ± 6.80	63.15 ± 6.68	63.25 ± 9.94	1–2	0.10	0.94
				2–3	−0.10	0.96
				1–3	0.00	1.00
HQR% Left Side	68.75 ± 8.49	68.10 ± 8.30	72.35 ± 10.35	1–2	0.65	0.74
				2–3	−4.25	0.07
				1–3	−3.60	0.14

Note: * *p* < 0.05. RQ: right quadriceps; LQ: left quadriceps; RH: right hamstring; LH: left hamstring; HQR%: hamstring-to-quadriceps ratio percentile.

**Table 5:** Cardiorespiratory measurements of the players at the beginning of the season (post-preseason), end of the regular season (postseason), and following the transition period (preseason) (Mean ± SD).

Parameter	Post-Preseason (1)	Postseason (2)	Preseason (3)	Repeated Measures ANOVA		
	(n = 17)	(n = 17)	(n = 17)		Mean Difference	Significance
Run time (min)	10.78 ± 1.70	9.75 ± 1.79	8.92 ± 1.82	1–2	1.02 *	<0.01
				2–3	0.83 *	<0.01
				1–3	1.86 *	<0.01
VO2max (mL/kg*min)	49.81 ± 4.16	48.17 ± 4.76	47.29 ± 4.64	1–2	1.64 *	<0.01
				2–3	0.88 *	<0.01
				1–3	2.53 *	<0.01
Velocity at VT1 (km/h)	9.53 ± 1.13	8.59 ± 1.18	8.24 ± 0.66	1–2	0.94 *	<0.01
				2–3	0.35	0.19
				1–3	1.29 *	<0.01
Velocity at VT2 (km/h)	11.53 ± 1.13	10.59 ± 1.37	10.24 ± 0.66	1–2	0.94 *	<0.01
				2–3	0.35	0.27
				1–3	1.29 *	<0.01
Velocity at VO2max (km/h)	13.18 ± 1.02	11.53 ± 1.13	11.53 ± 1.13	1–2	1.65 *	<0.01
				2–3	0.00	1.00
				1–3	1.65 *	<0.01

Note: * *p* < 0.05. VO2max: maximal oxygen uptake; VT1: first ventilatory threshold; VT2: second ventilatory threshold.

## Data Availability

Data can become available upon request.
